# Patient-initiated violence against dental staff: a survey in faculty clinic settings

**DOI:** 10.3389/fpubh.2025.1630346

**Published:** 2025-07-08

**Authors:** Avia Fux-Noy, Oriane Getter, Aviv Shmueli, Elinor Halperson, Moti Moskovitz

**Affiliations:** ^1^Faculty of Dental Medicine, Hebrew University of Jerusalem, Jerusalem, Israel; ^2^Department of Pediatric Dentistry, Hadassah Medical Center, Jerusalem, Israel

**Keywords:** workplace violence, patient-initiated violence, dental staff, dentists, survey

## Abstract

**Background:**

Workplace violence against healthcare personnel is an increasing concern. However, there is limited research on this issue within the dental field.

**Aim:**

This study aimed to examine the prevalence and characteristics of patient-initiated violence against dental staff.

**Methods:**

A cross-sectional survey design was utilized, involving a convenience sample of dental clinic staff at Hadassah Medical Center, Jerusalem, Israel. Participants filled out an anonymous questionnaire that assessed patient aggression in three categories: physical violence, verbal abuse, and reputational harm. Additionally, demographic information such as gender, age, role, and years of experience was collected.

**Results:**

The response rate was 29%. Of the 103 respondents, 73% were females, 79% were dentists; 95% reported experiencing verbal violence, 27% physical violence, and 53% reputational harm at least once in their career. Male staff reported significantly higher rates of reputational harm compared to female staff (*p* = 0.025). Dentists experienced significantly more reputational harm than dental auxiliaries (*p* = 0.004). No significant differences were found based on clinic specialization or years of experience.

**Conclusion:**

Dental clinic staff frequently experience high levels of verbal, physical, and reputational violence. It is essential to conduct larger, nationally representative studies in Israel to confirm these findings. Future research should examine the causes and consequences of patient-initiated violence and explore effective prevention and intervention strategies.

## 1 Introduction

The World Health Organization defines workplace violence as the intentional use of power, either threatened or actual, against an individual or group in work-related circumstances, resulting in or likely to result in injury, death, psychological harm, maldevelopment, or deprivation (1). Violence against medical staff is a recognized global issue and a form of workplace violence. Healthcare and social service industries experience the highest rates of workplace violence injuries, being five times more likely to suffer such injuries than workers in other sectors. The overall incidence of workplace violence has increased in recent years (2). Healthcare workers are four times more likely to be absent from work due to violence directed at them (3).

Violence against medical staff leads to significant negative consequences, including lower self-esteem, heightened anxiety and stress, decreased work performance, and a decline in the quality of patient care. Furthermore, it is linked to burnout, lower job satisfaction, compromised patient safety, increased medical errors, and higher rates of absenteeism and professional turnover (4).

Although numerous studies have documented violence against medical staff (5, 6), research on violence specifically targeting dental teams remains limited. Studies indicate that 29–80% of dentists have experienced workplace violence, primarily from patients, but also from patients’ relatives, colleagues, and supervisors (7–10). Dental procedures can evoke negative emotions such as fear, pain, anger, and mistrust in patients, which may lead to violent reactions. Several factors contribute to violence against dental staff, including long wait times, appointment cancellations, high treatment costs, and perceived unsatisfactory outcomes of treatment (11).

The Israeli dental care system comprises both private and public clinics, with certain procedures subsidized under the National Health Insurance Law. Due to the lack of previous surveys on violence directed at dental staff in Israel, this study aimed to assess the extent and nature of violence experienced by dental professionals. For these preliminary results, the investigation centered on the staff of the Faculty of Dental Medicine. It also explored potential associations between clinic specialization, staff roles, years of experience, and the frequency of reported incidents of violence.

## 2 Materials and methods

This study utilized a cross-sectional survey design using a convenience sample of dental clinic faculty staff. A convenience sample offers easy access to participants, speeds up data collection, and is appropriate for preliminary research or experiments aimed at testing basic assumptions (12). Ethical approval was obtained from the Institutional Review Board (HMO-0280-22).

### 2.1 Study population

Eligible participants were dental staff members at the Faculty of Dental Medicine, Hadassah Medical Center, including dentists, dental assistants, dental hygienists, and receptionists. Inclusion criteria were: providing patient care for at least 1 year and fluency in Hebrew (reading and speaking). Individuals not meeting these criteria were excluded. Of the 350 dental staff members at the faculty, 103 participants from seven departments responded to the questionnaire, yielding a response rate of 29%. With a sample size of 103, assuming a 95% confidence level and an estimated prevalence of 50%, the margin of error is approximately ±9.6%. While this margin of error is relatively high, it remains acceptable for generating useful preliminary insights, particularly in exploratory research (13).

### 2.2 Study instrument

The survey instrument, adapted from Rhoades et al. (10, 14), was translated into Hebrew using a forward-backward translation method by two independent translators fluent in both languages. Initially, a native Hebrew speaker with expertise in English translated the instrument into Hebrew. Then, a native English speaker conducted the back-translation. The back-translation was reviewed by the translators and authors, who confirmed there were no discrepancies in wording. This anonymous questionnaire assessed patient aggression across three categories: physical violence (9 items: kicked you, grabbed you, slapped you, pushed or shoved you, threw something at you, hit you, twisted your arm or hair, damage or attempt to damage property in or around your practice, threatened you with a weapon), verbal violence (8 items: raised their voice angrily at you, insulted you, used foul language toward you, threatened to hit or throw something at you, used derogatory language regarding your gender/race/ethnicity/sexual orientation/age, called you a demeaning name, threatened to physically harm you, harassed you via the phone, Internet, or text message), and reputational harm (4 items: threatened to post nasty comments about you and/or your practice on the Internet or something similar, reported you to a licensing body or government agency (without cause), threatened to report you to a licensing body or government agency (without cause), threatened to sue you and/or your practice). Participants indicated the frequency of each experience using the following scale: “never,” “not this year, but in the past,” “once in the last year,” “twice in the last year,” and “three or more times in the last year.” Demographic data, including participant’s gender, age, role, and years of experience, were also collected. Both digital and paper versions of the survey were distributed to eligible participants. Anonymity was ensured by not collecting any identifying information and by storing responses using numeric codes.

### 2.3 Statistical analysis

For statistical analysis, participants were categorized as having experienced violence within a specific category if they reported at least one instance of that type of violence. To facilitate meaningful statistical analysis and ensure sufficient sample size within each subgroup, staff roles were grouped into three categories: (1) assistants, hygienists, and receptionists; (2) general dentists and residents; and (3) specialist dentists. This classification was informed by both the functional similarities within each group and their distinct levels of clinical responsibility, patient interaction, and authority in the dental setting. Frequency of violence exposure was condensed into three categories: (0) never, (1) not this year, but in the past, and (2) once or more in the last year. Condensing into three broader groups helps maintain sufficient numbers within each category to enable robust statistical analysis. Data was analyzed using SPSS software to examine relationships between violence type, frequency, and demographic variables. Chi-square tests or Fisher’s exact tests and logistic regression were used to evaluate these relationships, with statistical significance set at *p* ≤ 0.05.

## 3 Results

The respondent pool comprised 27% males and 73% females. Table 1 details the participants’ departmental affiliation, role within the clinic and years of experience. Table 2 presents the results regarding the type and frequency of violence experienced. When asked about the location of violent incidents, 67% of respondents reported occurrences in the faculty clinic, 36% in a public clinic, and 20% in a private clinic.

**Table 1:** Demographic data distribution.

Department [n(%)]	Role [n(%)]	Years of experience [n(%)]
Pedodontic 30(29.1)	General dentist 4(4)	1–5 years 31(30)
Periodontic 15(14.6)	Resident dentist 44(43)	6–10 years 30(29)
Oral medicine 12(11.7)	Specialist dentist 33(32)	11–15 years 10(10)
Prosthodontic 13(12.6)	Assistant 16(15)	16–20 years 11(11)
Endodontic 10(9.7)	Hygienist 1(1)	Over 20 years 21(20)
Orthodontic 14(13.6)	Receptionist 5(5)	
Maxillofacial surgery 9(8.7)		

**Table 2:** Violence type and frequency.

Violence category	Yes		Once or more in the last year		Not this year, but in the past	
	Proportion (%)	95% Confidence Interval	Proportion (%)	95% Confidence Interval	Proportion (%)	95% Confidence Interval
Verbal violence	95%	89.1–97.9%	74%	64.5–81.3%	26%	18.7–35.5%
Physical violence	27%	19.5–36.5%	57%	47.6–66.4%	43%	33.6–52.4%
Reputational harm	53%	43.8–62.7%	42%	32.7–51.4%	58%	48.6–67.3%

**Figure 1:**
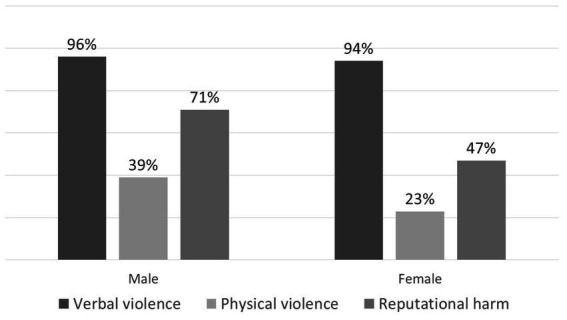
Violence type according to participant’s sex.

Analysis of the relationship between staff gender and the type and frequency of violence (Figure 1) revealed that male staff members reported higher rates of physical violence and reputational harm compared to female staff members. Specifically, a statistically significant difference was observed in reports of reputational harm (*p* = 0.025).

**Figure 2:**
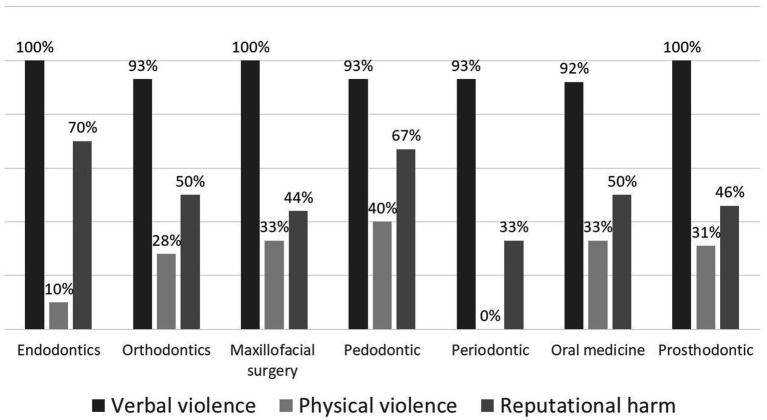
Violence type according to participant’s department.

Analysis of the relationship between departmental affiliation and violence type and frequency (Figure 2) indicated that staff in the pedodontics department reported higher rates of physical violence compared to other departments, though this difference was not statistically significant (*p* = 0.066). Similarly, staff in the endodontic and pedodontics departments reported higher rates of reputational harm, but this difference also lacked statistical significance (*p* = 0.390).

**Figure 3:**
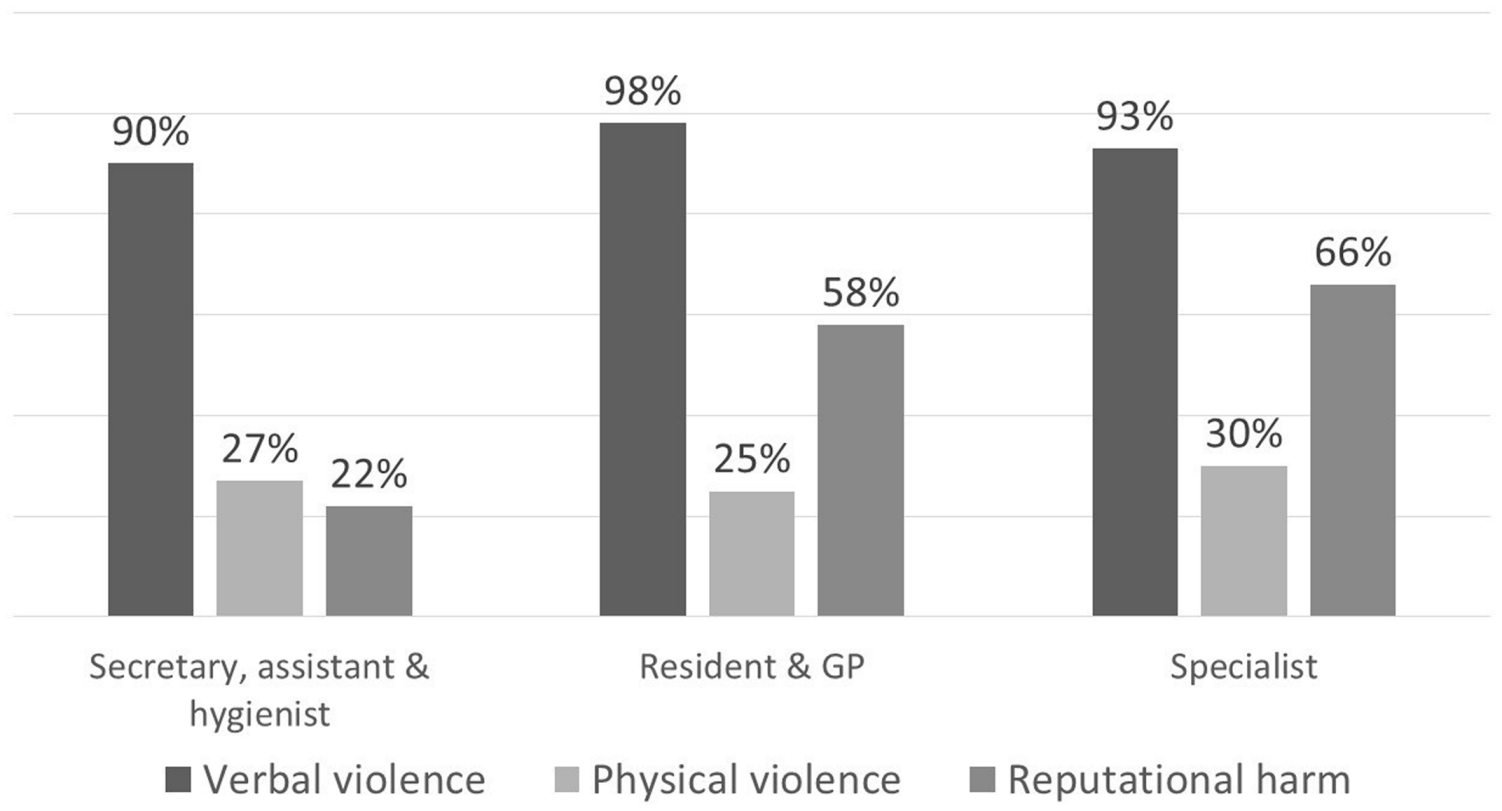
Violence type according to participant’s role.

Analysis of the relationship between staff role and violence type and frequency (Figure 3) revealed that specialist dentists, general dentists and residents reported significantly higher rates of reputational harm compared to dental assistants and receptionists (*p* = 0.004).

**Figure 4:**
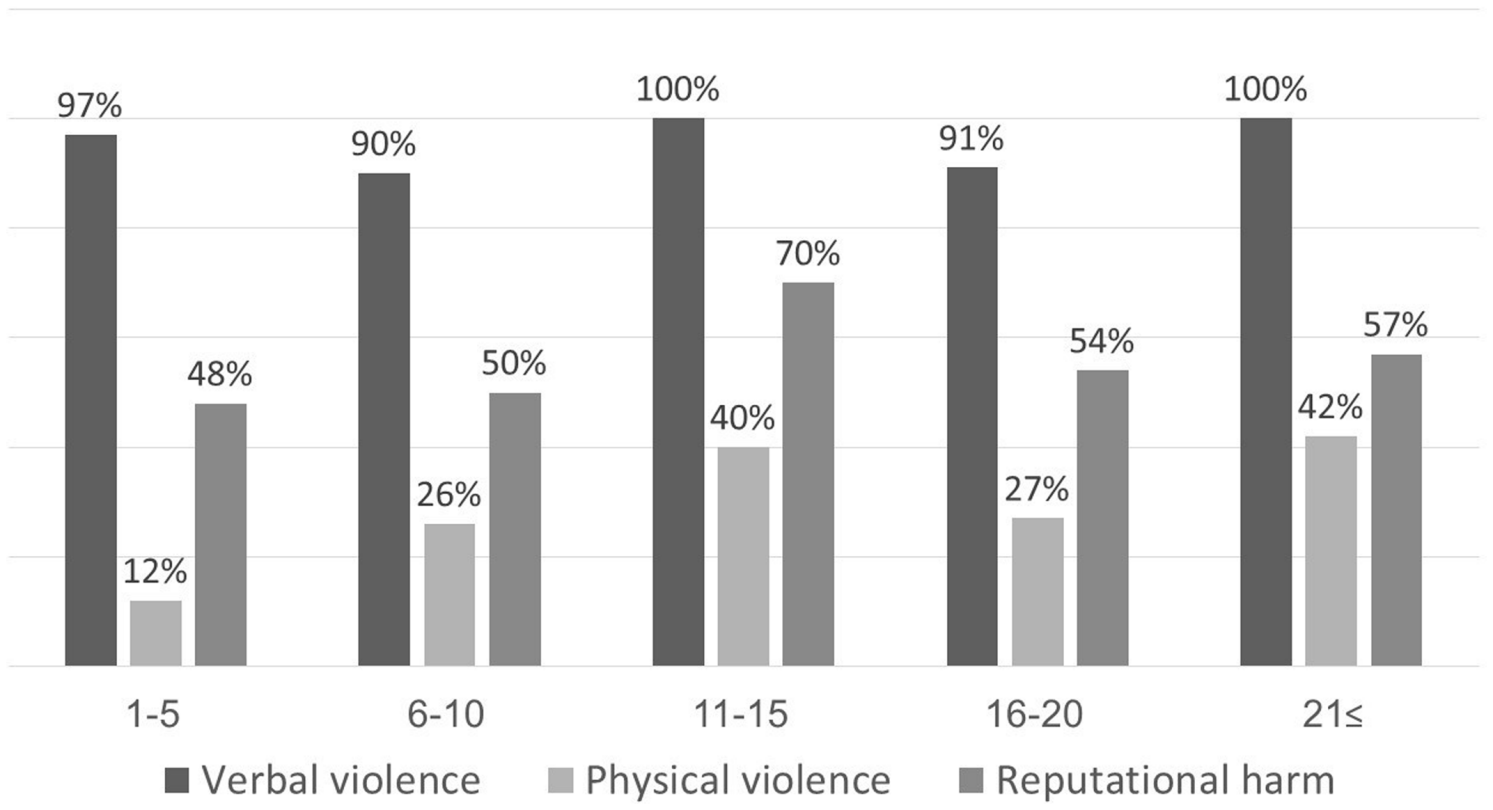
Violence type according to participant’s years of experience.

Analysis of the relationship between years of experience and the type and frequency of violence (Figure 4) showed an increasing trend in reported physical violence and reputational harm as experience increased. A linear relationship was observed between physical violence and experience (linear variables *p* = 0.023), however, this did not reach statistical significance using Fisher’s exact test (*p* = 0.133). Similarly, no statistically significant association was found between years of experience and reputational harm (*p* = 0.809). Additionally, no consistent relationship was found between years of experience and the occurrence of recent violence. (i.e., within the last year vs. in the past).

To examine factors linked to patient-initiated violence, including verbal, physical, or reputational separately, a logistic regression model was fitted with role, department, gender, age, and years of experience as predictors (Table 3). The results indicate that an individual’s role is an important predictor of whether they experience reputational harm, with an odds ratio of 2.112 (*p* = 0.022). This suggests that specialist dentists have more than twice the odds of experiencing reputational damage compared to auxiliary staff.

**Table 3:** Logistic regression models predicting each type of patient-initiated violence: verbal, physical, and reputation damage.

Type of patient-initiated violence	Predictor	Odds ratio (OR)	*p*-value	95% CI (lower–upper)
Verbal	Intercept	0.028	0.537	0.000–2291.713
	Role	0.804	0.796	0.154–4.200
	Department	1.044	0.877	0.604–1.805
	Gender	1.468	0.771	0.111–19.491
	Age	1.252	0.185	0.898–1.745
	Experience	0.381	0.275	0.067–2.158
Physical	Intercept	0.412	0.687	0.006–30.786
	Role	0.778	0.455	0.402–1.504
	Department	1.023	0.867	0.787–1.328
	Gender	0.507	0.225	0.169–1.518
	Age	1.025	0.610	0.932–1.128
	Experience	1.177	0.635	0.601–2.305
Reputation	Intercept	0.362	0.638	0.005–25.108
	Role	2.112	0.022	1.114–4.007
	Department	0.888	0.334	0.699–1.129
	Gender	0.554	0.277	0.191–1.606
	Age	1.046	0.354	0.951–1.150
	Experience	0.757	0.399	0.397–1.444

## 4 Discussion

The study’s findings indicate a considerable occurrence of violence against staff in dental clinics. Notably, 95% of participants reported experiencing verbal violence, 27% reported physical violence, and 53% reported reputational harm at least once, either in the past year or at some point previously.

The existing literature on violence against dental staff comprises studies that either focus on specific professional groups, such as dentists (9, 10, 15), dental hygienists (16, 17), or dental students (14, 18, 19), or examine the entire staff (8), as was the approach in our study. The differences in scope make it difficult to directly compare the results of various studies. The methodology used in the current study was adapted from Rhoades et al. (10, 14). In comparison to our findings, Rhoades et al. reported prevalence rates of 74% for verbal violence, 45.5% for physical violence, and 68.7% for reputational harm among dentists (10), and 86% for verbal violence, 28% for physical violence, and 36% for reputational harm among dental students (14). A recent systematic review and meta-analysis on the prevalence of violence against oral healthcare workers reported that verbal abuse ranged from 8.2 to 58.7%, and physical abuse ranged from 4.6 to 22%. The review also addressed sexual harassment, with prevalence rates ranging from 6.8 to 54% in nearly all included studies (11).

While the current study revealed a significant difference in reported reputational harm, with dentists experiencing higher rates compared to auxiliary staff, Azodo et al. (8) found no such difference in the overall prevalence of violence between these groups. This discrepancy may be attributed to methodological variations, since the current questionnaire’s distinct categorization of physical, verbal, and reputational violence. The observed difference in our study was limited to reputational harm, a form of violence likely to be disproportionately directed towards dentists compared to assistants and receptionists. Dentists bear direct responsibility for diagnosis, treatment planning, and clinical outcomes. This central role places them at the forefront of patient interactions and expectations. When patients are dissatisfied, they may attribute blame to the dentist, who is seen as the decision-maker. This makes dentists more likely targets of reputational harm, as patients may express frustration through complaints or social media criticism. In contrast, assistants and receptionists typically serve in supporting roles and have limited influence over clinical decisions, making them less likely to be held personally accountable for patient dissatisfaction. This structural dynamic within the dental care team likely contributes to the observed differences in reputational harm reported across roles. The grouping approach may mask subtle differences between, for instance, dental hygienists and receptionists within the auxiliary category, or between residents and general dentists. However, the classification strikes a balance between analytical feasibility and professional relevance, making it suitable for identifying general trends while acknowledging some granularity is lost.

Our survey results indicate that male staff members reported higher rates of physical violence and reputational harm compared to female staff members. This finding is consistent with a research report conducted among community health workers in Israel (20), which also reported higher rates of physical violence against men and comparable rates of verbal violence between genders. However, Rhoades et al. (10), in a study of dentists, found no association between gender and reported experiences of violence. In Israeli society, similar to many cultures, male healthcare professionals are frequently viewed as authority figures. This perception can lead patients to express their dissatisfaction or frustration toward male staff. Additionally, males are generally more likely to display and report aggressive behaviors and may adopt more direct or assertive communication styles. Such styles can be perceived by patients as dismissive or authoritarian, potentially escalating tensions during clinical interactions. Furthermore, males may be more likely to interpret and report certain negative encounters as reputational damage, rather than as emotional or indirect aggression (21, 22).

Our findings indicate a higher incidence of physical and reputational violence reported in the pediatric dentistry clinic compared to other departments. However, these results should be interpreted cautiously due to the disproportionately higher response rate from the pedodontics department, which was two to three times that of other departments. This overrepresentation may skew the findings by giving disproportionate weight to experiences unique to the pediatric dentistry department. While the data suggest a potential difference in violence exposure compared to other departments, the uneven response rates limit our ability to draw definitive conclusions or generalize these findings across the broader dental staff population. Furthermore, pediatric dentistry in Israel, being nationally funded, experiences a significantly increased workload and high patient demand, which may further differentiate its working conditions from those in other specialties. To accurately identify trends in violence against dental staff across different specializations, a larger study with equitable representation from all departments is necessary.

It is crucial to investigate further implications of the findings. Exposure to workplace violence has been consistently associated with decreased job satisfaction, increased burnout, workforce attrition, and psychological distress among dental healthcare providers. Moreover, it may compromise the quality of patient care (4). Providers may experience reduced focus, diminished empathy, all of which can undermine clinical performance and communication. This may erode trust between patients and providers, an essential component of effective dental care. Therefore, the findings underscore the need for institutional policies that proactively address workplace violence, including training, reporting mechanisms, and psychological support.

This study has several limitations. First, the response rate was relatively low (29%), with a final sample size of 103 participants. Although low, the expected response to an online questionnaire has been described as 25–30% (23). Such a response rate introduces a risk of nonresponse bias, whereby the dentists who chose to participate may differ systematically from those who did not, potentially in their experiences, perceptions, or willingness to disclose incidents of patient-initiated violence. This could lead to an overestimation or underestimation of the true prevalence or impact of such experiences in the broader dental workforce. Second, the small sample size drawn from a single medical center in Israel, limits the statistical power to detect subtle differences or associations and restricts the generalizability of the findings. It also limits the ability to conduct meaningful subgroup analyses within the sample. This study served as an initial exploration of patient-initiated violence in dental clinics. The results should therefore be interpreted with caution and considered exploratory. Third, the use of convenience sampling and voluntary participation may have further compounded selection bias, as dentists with strong opinions or personal experiences related to patient-initiated violence may have been more motivated to respond. Future research in this area would benefit from employing strategies to improve response rates, such as multiple follow-up contacts, offering participation incentives, or using mixed methods approaches. Additionally, larger, randomly selected samples would enhance the representativeness and reliability of findings on this important occupational health issue. Furthermore, the study did not explore specific details of violent incidents, including their triggers, the effects on staff members (such as physical injury or psychological trauma), or how they were resolved. Future research should focus on these details to better comprehend the context of violent events and develop effective intervention strategies.

In conclusion, dental clinic staff frequently experience high levels of verbal, physical, and reputational violence. It is essential to conduct larger, nationally representative studies in Israel to confirm these findings. Future research should examine the causes and consequences of patient-initiated violence and explore effective prevention and intervention strategies.

## Data Availability

The original contributions presented in the study are included in the article/supplementary material, further inquiries can be directed to the corresponding author.

## References

[1] Cooper C Swanson N. Workplace violence in the health sector. State of the Art (2002). Available online at: https://www.who.int/docs/default-source/documents/violence-against-health-workers/wvstateart.pdf?sfvrsn=36aae706_2 (Accessed February 28, 2025).

[2] U.S. Bureau of Labor statistics. Fact sheet. Workplace Violence in Healthcare (2018). Available online at: https://www.bls.gov/iif/factsheets/workplace-violence-healthcare-2018.htm (Accessed February 28, 2025).

[3] Phillips JP. Workplace violence against health care workers in the United States. N Engl J Med. (2016) 374:1661–9. doi: 10.1056/NEJMra1501998, PMID: 27119238

[4] Sahebi A Golitaleb M Moayedi S Torres M Sheikhbardsiri H. Prevalence of workplace violence against health care workers in hospital and pre-hospital settings: an umbrella review of meta-analyses. Front Public Health. (2022) 10:895818. doi: 10.3389/fpubh.2022.895818, PMID: 36003634 PMC9393420

[5] Aljohani B Burkholder J Tran QK Chen C Beisenova K Pourmand A. Workplace violence in the emergency department: a systematic review and meta-analysis. Public Health. (2021) 196:186–97. doi: 10.1016/j.puhe.2021.02.009, PMID: 34246105

[6] Li YL Li RQ Qiu D Xiao SY. Prevalence of workplace physical violence against health care professionals by patients and visitors: a systematic review and Meta-analysis. Int J Environ Res Public Health. (2020) 17:299. doi: 10.3390/ijerph17010299, PMID: 31906306 PMC6982349

[7] Pemberton MN Atherton GJ Thornhill MH. Violence and aggression at work. Br Dent J. (2000) 189:409–10. doi: 10.1038/sj.bdj.4800785, PMID: 11093388

[8] Azodo CC Ezeja EB Ehikhamenor EE. Occupational violence against dental professionals in southern Nigeria. Afr Health Sci. (2011) 11:486–92. PMID: 22275944 PMC3261011

[9] Ayers KM Thomson WM Newton JT Morgaine KC Rich AM. Self-reported occupational health of general dental practitioners. Occup Med (Lond). (2009) 59:142–8. doi: 10.1093/occmed/kqp004, PMID: 19223433

[10] Rhoades KA Heyman RE Eddy JM Haydt NC Glazman JE Dispirito ZF. Patient aggression toward dentists. J Am Dent Assoc. (2020) 151:764–9. doi: 10.1016/j.adaj.2020.06.041, PMID: 32979955

[11] Binmadi NO Alblowi JA. Prevalence and policy of occupational violence against oral healthcare workers: systematic review and meta-analysis. BMC Oral Health. (2019) 19:1–8. doi: 10.1186/s12903-019-0974-3 31830978 PMC6909447

[12] Etikan I Musa SA Alkassim RS. Comparison of convenience sampling and purposive sampling. Am J Theor Appl Stat. (2016) 5:1–4. doi: 10.11648/j.ajtas.20160501.11

[13] Hertzog MA. Considerations in determining sample size for pilot studies. Res Nurs Health. (2008) 31:180–91. doi: 10.1002/nur.20247, PMID: 18183564

[14] Rhoades KA Heyman RE Eddy JM Fat SJ Haydt NC Glazman JE. Patient aggression toward dental students. J Dent Educ. (2020) 84:586–92. doi: 10.1002/jdd.12044, PMID: 32022267

[15] Mannion CJ Gordon C. Aggression directed towards members of the oral and maxillofacial surgical team. Br J Oral Maxillofac Surg. (2018) 56:482–5. doi: 10.1016/j.bjoms.2018.01.015, PMID: 29885985

[16] Petit JN Boyd LD Vineyard J Dominick C. A survey of the prevalence and predictors of workplace bullying towards the dental hygienist. Int J Dent Hyg. (2021) 19:332–9. doi: 10.1111/idh.12493, PMID: 33756066

[17] Ghoneim A Parbhakar KK Farmer J Quiñonez C. Healthy and respectful workplaces: the experiences of dental hygienists in Canada. JDR Clin Trans Res. (2022) 7:194–204. doi: 10.1177/23800844211001827, PMID: 33754872

[18] Khanagar SB Aldawas I Almutairi A Alamro M Altammami N Aldakhil S. Dental students' experience, impact, and response to patient aggression in Saudi Arabia: a nationwide study. Healthcare (Basel). (2022) 10:2239. doi: 10.3390/healthcare10112239, PMID: 36360580 PMC9690685

[19] Looper A Esfandiari S. The prevalence of patient aggression toward dental students at a Canadian university teaching clinic. J Can Dent Assoc. (2023) 89:n4 37562037

[20] Nagar G Hazan-Hazoref R. Violence against staff in community clinics: May 2021 research report. [Hebrew] Available online at: www.gov.il/BlobFolder/reports/violence-medical-staff-report-052021/he/publications_science_violence-against-medical-staff-may-2021-accessible.pdf (Accessed February 28, 2025).

[21] Tziner A Bar-Mor H Shwartz-Asher D Shkoler O Geva L Levi H. Insights into abusive workplace behavior. Front Psychol. (2023) 14:990501. doi: 10.3389/fpsyg.2023.990501, PMID: 37575441 PMC10421746

[22] Björkqvist K. Sex differences in physical, verbal, and indirect aggression: a review of recent research. Sex Roles. (1994) 30:177–88. doi: 10.1007/BF01420988

[23] Fincham JE. Response rates and responsiveness for surveys, standards, and the journal. Am J Pharm Educ. (2008) 72:43. doi: 10.5688/aj720243, PMID: 18483608 PMC2384218

